# Integrated metabolome and transcriptome analysis reveals the potential mechanism of anthocyanin biosynthesis in *Paeonia lactiflora* Pall. flowers

**DOI:** 10.3389/fpls.2026.1907085

**Published:** 2026-08-31

**Authors:** Yang Cao, Pin Lv, Yongchuan Guo, Rui Deng, Yanxia Lv, Chuanling Zhang, Yan Xue, Xin Jia, Peifeng Xue, Chaoqun Xu, Xiaoqin Wang

**Affiliations:** 1 College of Pharmacy, Inner Mongolia Medical University, Hohhot, China; 2 Ethnic Medicine Innovation Center, Inner Mongolia Medical University, Hohhot, China; 3 Inner Mongolia Autonomous Region Agricultural and Animal Husbandry Technology Promotion Center, Hohhot, China; 4 Institute of Medicinal Plant Development, Chinese Academy of Medical Sciences & Peking Union Medical College, Beijing, China

**Keywords:** anthocyanin, metabolome, *Paeonia lactiflora* Pall., petal, transcriptome

## Abstract

*Paeonia lactiflora* Pall. (Chinese peony), a perennial plant indigenous to northeastern China, exhibits extensive flower-color variation, but the metabolite-gene relationships underlying anthocyanin accumulation remain incompletely understood. We integrated metabolomic and transcriptomic of petals from three cultivars with pink (cv. ‘Exclusive Memory’, PF), pale pink (cv. ‘Salad’, PPF), and white (cv. ‘Duchess’, WF) flowers. Metabolomic profiling detected 689 metabolites, including 169 flavonoids and six anthocyanins. Pairwise comparisons identified 153, 199, and 137 differentially accumulated metabolites in PF *vs.* PPF, PF *vs.* WF, and PPF *vs.* WF, respectively, with 27 metabolites shared among the three comparisons. The total anthocyanin signal in PF petals was 3.25-fold that in PPF petals and 60.37-fold that in WF petals. By contrast, the abundance of the individual compound cyanidin-3,5-*O*-diglucoside (cyanin) was 76.85-fold higher in PF than in WF and 23.10-fold higher in PPF than in WF, these values therefore describe compound-specific rather than total-anthocyanin differences. RNA-seq identified 6,777, 8,030, and 6,794 differentially expressed genes in the three pairwise comparisons, including 779 shared genes. Correlation analysis prioritized 21 flavonoid-pathway structural genes and 53 candidate transcription factors associated with anthocyanin abundance. Weighted gene co-expression network analysis identified a turquoise module positively associated with anthocyanins and a *DFR*-centered subnetwork containing 24 candidate transcription factors, including *MYB* and *bHLH* genes. qRT-PCR of 12 selected genes reproduced the RNA-seq expression trends. These results elucidate the metabolic basis of peony petal coloration and identify candidate regulatory networks and key genes potentially involved in anthocyanin biosynthesis, providing a foundation for future functional validation.

## Introduction

*Paeonia lactiflora* Pall. (Chinese peony) is an economically important ornamental and medicinal species with cultivars that display marked variation in petal color. Flavonoids, particularly anthocyanins, contribute substantially to this diversity, and cultivar-dependent differences in floral metabolite composition have been reported in *P. lactiflora* (Hafeez et al., 2022; Li et al., 2024). Identifying the metabolites and expressed genes associated with color intensity is therefore relevant to both germplasm evaluation and flower-color breeding.

Plant coloration is determined by the composition, modification, concentration, and cellular environment of pigments. Anthocyanidins are commonly grouped into six major aglycone classes-cyanidin, pelargonidin, delphinidin, peonidin, petunidin, and malvidin-and their glycosylated and acylated derivatives generate colors ranging from orange-red to purple-blue (Wang et al., 2021; Li et al., 2022). Other flavonoid subclasses, including chalcones, flavones, flavonols, flavanones, flavanols, and flavanonols, can also influence coloration through co-pigmentation and competition for shared precursors (Cho et al., 2016). Integrated metabolomic studies have identified color-associated flavonoids in sweet potato, safflower, and *Camellia japonica* (Wang et al., 2018, Wang et al., 2021, Wang et al., 2023; Fu et al., 2021). In *C. japonica*, for example, cyanidin-3-*O*-(6’’-*O*-malonyl) glucoside was associated with red petals (Fu et al., 2021). These findings emphasize that both total pigment abundance and the identities of individual anthocyanins must be considered when explaining flower color.

Anthocyanins are produced through the phenylpropanoid-flavonoid pathway (Chen et al., 2020). Early structural enzymes, including phenylalanine ammonia-lyase (*PAL*), 4-coumarate:CoA ligase (*4CL*), chalcone synthase (*CHS*), and chalcone isomerase (*CHI*), supply flavonoid precursors, whereas dihydroflavonol 4-reductase (*DFR*), anthocyanidin synthase (*ANS*), and glycosyltransferases catalyze late steps leading to stable anthocyanins (He et al., 2020; Permyakov et al., 2013). Functional and multi-omics studies in several species have linked altered expression of these genes to anthocyanin accumulation (Mierziak et al., 2014; Xia et al., 2022). Their transcription is commonly coordinated by *MYB*, *bHLH*, and *WD40* proteins, while *WRKY*, *NAC*, *bZIP*, and other transcription-factor families may provide additional regulatory inputs (Lloyd et al., 2017; Ding et al., 2025; Wang et al., 2024; Li et al., 2023). However, the candidate genes and co-expression relationships associated with graded anthocyanin accumulation among *P. lactiflora* cultivars remain insufficiently resolved.

Integrated metabolomic and transcriptomic analyses can connect changes in pigment abundance with changes in gene expression and thereby prioritize candidate components of a biosynthetic network (Fu et al., 2021; Lu et al., 2021). Here, we compared three *P. lactiflora* cultivars representing a pink-to-white color gradient using matched metabolomic and RNA-seq samples. We aimed to (i) identify anthocyanins associated with petal color intensity, (ii) characterize differentially expressed structural genes and transcription factors in the flavonoid pathway, and (iii) use metabolite-gene correlations and WGCNA to nominate co-expression modules and hub candidates. This study serves as a reference for understanding the molecular mechanisms underlying the precise control of anthocyanin biosynthesis and accumulation in peony.

## Materials and methods

### Plant materials and sample preparation

Three *P. lactiflora* cultivars were grown in the Medicinal Botanical Garden of Inner Mongolia Medical University, Hohhot, Inner Mongolia Autonomous Region, China (40°51′ N, 111°46′ E). The cultivars were cv. ‘Exclusive Memory’ (PF), cv. ‘Salad’ (PPF), and cv. ‘Duchess’ (WF). Fully opened flowers were collected in May 2022 at the full-bloom stage. For each cultivar, three independent biological replicates were collected. Each biological replicate consisted of petals obtained from six independent plants and six flowers and petals were pooled. No technical replicate was treated as an independent sample in the metabolomic or RNA-seq analyses. Each biological sample was divided into aliquots for matched metabolomic and transcriptomic profiling. Petals were immediately frozen in liquid nitrogen and stored at −80 °C until analysis.

### Metabolite extraction and profiling

Frozen petals were ground for 90 s at 30 Hz in a mixer mill (MM 400, Retsch) with a zirconia bead. Approximately 100 mg of powder was extracted overnight at 4 °C in 1.0 mL of 70% aqueous methanol (v/v). After centrifugation at 12,000 rpm for 10 min, the supernatant was filtered (ANPEL, Shanghai, China) and analyzed using a SHIMADZU Nexera X2 UPLC system coupled to an electrospray-ionization triple quadrupole-linear ion trap mass spectrometer. Chromatographic separation was performed on an Agilent SB-C18 column (1.8 μm, 2.1 mm × 100 mm) at 40 °C with a flow rate of 0.35 mL min^-1^ and an injection volume of 4 μL. Mobile phase A was water containing 0.1% formic acid, and mobile phase B was acetonitrile containing 0.1% formic acid. The gradient was 95:5 A:B at 0 min, changed linearly to 5:95 A:B over 9 min, and was maintained for 1 min. The ion-source temperature was 550 °C, and the ion-spray voltages were 5,500 V in positive mode and −4,500 V in negative mode. Gas 1, gas 2, and curtain gas were set to 50, 60, and 25 psi, respectively. Collision-activated dissociation was set to high, and multiple-reaction-monitoring scans were acquired with nitrogen as the collision gas.

### Metabolome data analysis

Metabolite extraction, identification, and quantification were performed by Metware Biotechnology Co., Ltd. (Wuhan, China) following the widely targeted UPLC-ESI-MS/MS workflow described by Wang et al. (2019). Quality-control samples were prepared by pooling equal aliquots of all biological samples and were analyzed throughout the run to evaluate analytical reproducibility.

Metabolomic analyses were conducted in R version 4.2.2. Principal component analysis was used to evaluate sample separation and within-group reproducibility. Orthogonal partial least-squares discriminant analysis (OPLS-DA) was used to calculate variable importance in projection (VIP) values, and 200 permutation tests were used to assess model overfitting. Differentially accumulated metabolites (DAMs) were defined by VIP ≥ 1 and fold change ≥ 2 or ≤ 0.5. DAMs were annotated using the Kyoto Encyclopedia of Genes and Genomes (KEGG) compound database and mapped to KEGG pathways. Pathway enrichment was evaluated by a hypergeometric test, with *P* ≤ 0.05 and false discovery rate (FDR) ≤ 0.05 considered significant.

### Transcriptome sequencing and data analysis

Base calling and demultiplexing were performed using CASAVA. Trimmomatic version 0.36 was used to remove adapter sequences and low-quality reads; reads containing at least five ambiguous bases or more than 50% low-quality bases were excluded. Q20, Q30, and GC content were calculated for the clean reads. Transcript abundance was quantified as fragments per kilobase of transcript per million mapped reads (FPKM). Differentially expressed genes (DEGs) were identified using DESeq2 version 1.34.0 with |log2(fold change)| ≥ 1 and Benjamini-Hochberg-adjusted FDR < 0.05. DEGs were functionally annotated using Gene Ontology (GO) and KEGG databases.

### Identification of structural genes and transcription factors

Candidate structural genes were selected from expressed transcripts annotated to phenylpropanoid biosynthesis (ko00940), flavonoid biosynthesis (ko00941), and flavone and flavonol biosynthesis (ko00944), together with similarity searches against NR and TAIR (E-value ≤ 1 × 10^−10^). Candidate transcription factors were annotated using PlantTFDB version 5.0 and iTAK version 2.0.1,.

### Metabolites-genes correlation and weighted gene co-expression network analysis

Pearson correlations were calculated between the abundance of anthocyanins and gene-expression values across the matched biological samples. Gene-metabolite pairs with |r| > 0.80 and *P* < 0.01 were retained as candidate associations. WGCNA was conducted in R using the WGCNA framework (Langfelder and Horvath, 2008). A soft-thresholding power of β = 18 was selected to obtain an approximate scale-free topology (R² > 0.85), and modules were identified from the topological overlap matrix using hierarchical clustering and dynamic tree cutting. Module eigengenes were correlated with anthocyanin abundance to identify color-associated modules. Candidate hub genes were prioritized using TOM weight > 0.24, |module membership| > 0.80, and |gene significance| > 0.20.

### Quantitative real-time PCR analysis

Total RNA was isolated using an RNA extraction kit (Monad Biotech, Wuhan, China) and treated with DNase I. qRT-PCR was performed using a CFX96 Touch Real-Time PCR Detection System (Bio-Rad, USA). Each 10 μL reaction contained 5 μL of 2× SYBR Green PCR Master Mix, 0.4 μL each of forward and reverse primer (10 μM), 1 μL of cDNA, and 3.2 μL of RNase-free water. Cycling conditions were 95 °C for 2 min, followed by 40 cycles of 95 °C for 10 s and 60 °C for 30 s, a melting-curve analysis was performed to verify amplification specificity. 12 genes were examined: *DFR* (Cluster-309990), *MYB* (Cluster-356500 and Cluster-367900), *4CL* (Cluster-297840), *CHI* (Cluster-443175 and Cluster-443178), *PAL* (Cluster-252400), *F3’5’H* (Cluster-212350), *FLS* (Cluster-281620) and *bHLH* (Cluster-437230, Cluster-298880, and Cluster-253660). *UBQ10* was used as the reference gene, and relative expression was calculated using the 2^^−ΔΔCt^ method. Three independent biological replicates were analyzed per cultivar, with three technical reactions per biological replicate. Primer sequences were provided in **Supplementary Table 1**.

### Statistical analysis

Statistical analyses were performed in R version 4.2.2. Metabolomic and RNA-seq analyses each used three independent biological replicates per cultivar. qRT-PCR used three independent biological replicates, with three technical reactions per biological replicate. Metabolite and transcriptome significance thresholds are described in their respective subsections, and FDR values were calculated using the Benjamini-Hochberg procedure. Pearson correlations were used for candidate prioritization and were not interpreted as causal evidence. Group differences in total anthocyanin abundance and qRT-PCR expression were evaluated using one-way ANOVA followed by Tukey’s HSD with *P* < 0.05 considered significant.

## Results

### Phenotypic and metabolomic characterization of three Chinese peony cultivars

**Figure 1:**
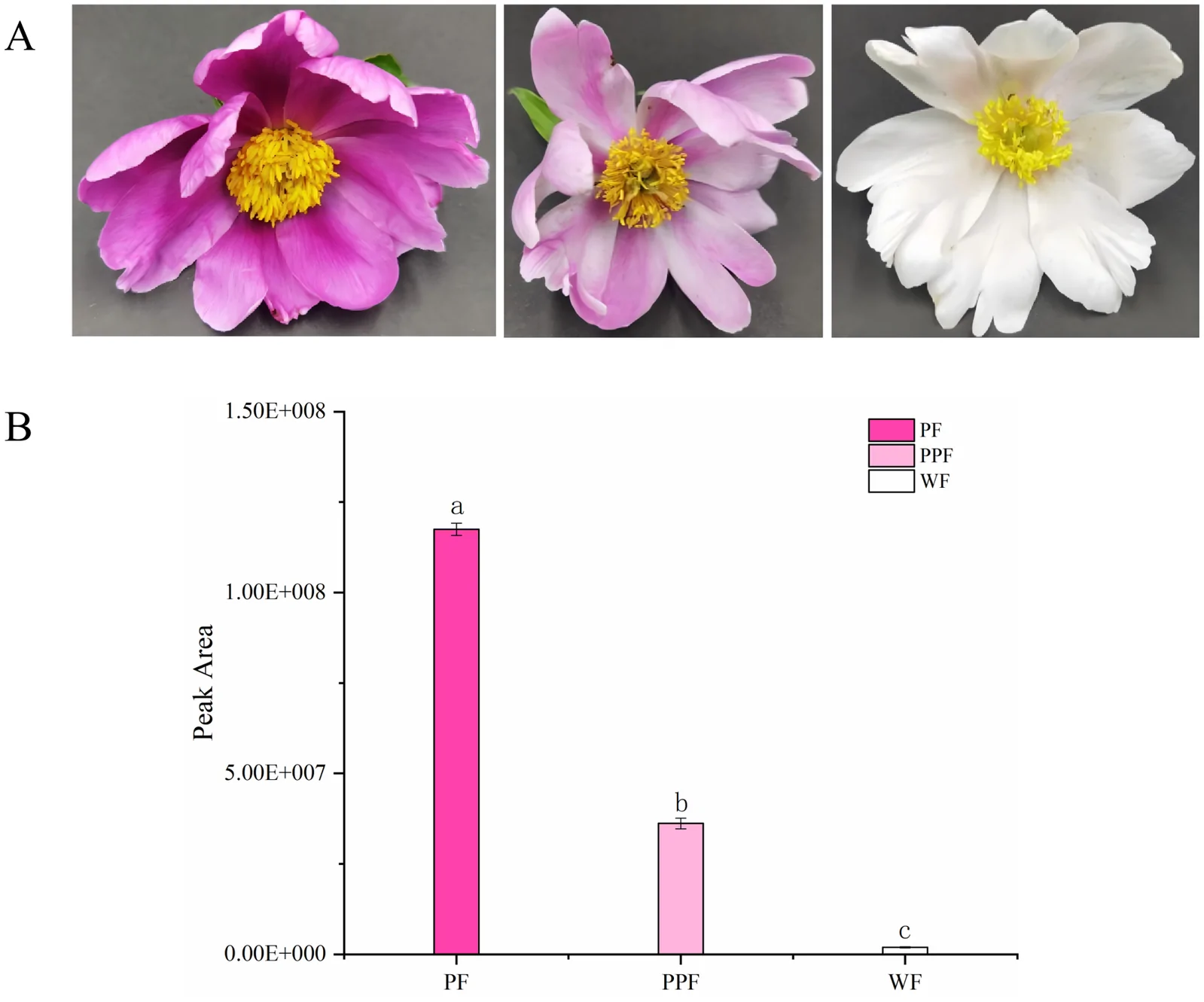
Petal phenotypes and total anthocyanin signal in three *P. lactiflora* cultivars. **(A)** Fully opened flowers of cv. ‘Exclusive Memory’ (PF), cv. ‘Salad’ (PPF), and cv. ‘Duchess’ (WF). **(B)** Total anthocyanin peak area in the three cultivars. Values are means ± SD of three biological replicates (n = 3). Different letters indicate significant differences at *P* < 0.05 according to one-way ANOVA followed by Tukey’s HSD.

**Figure 2:**
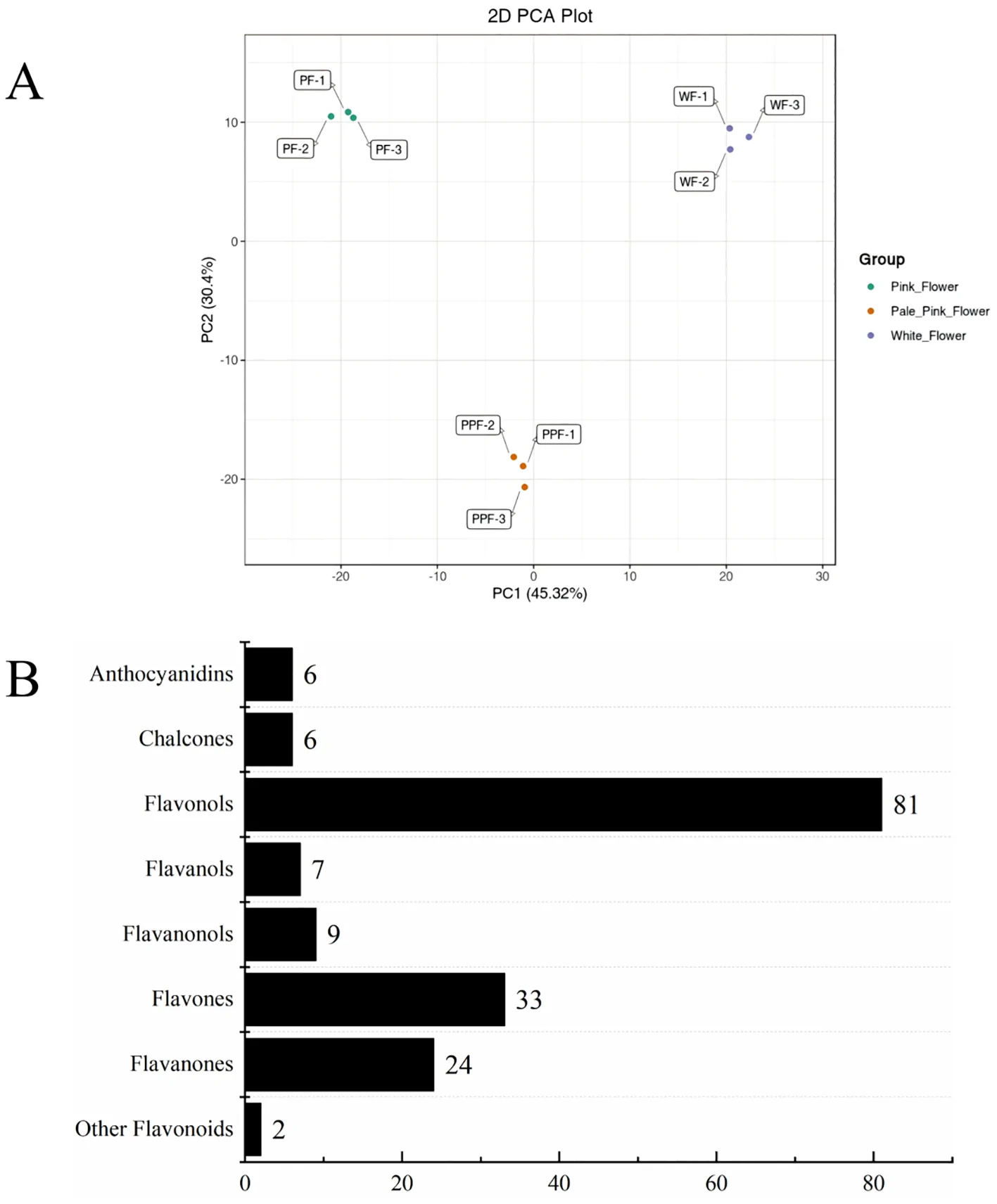
Overview of the metabolomic data. **(A)** Principal component analysis of metabolite abundance in PF, PPF, and WF petals. Each point represents one biological replicate. **(B)** Classification of the 689 detected metabolites, including the detailed flavonoid subclasses.

**Figure 3:**
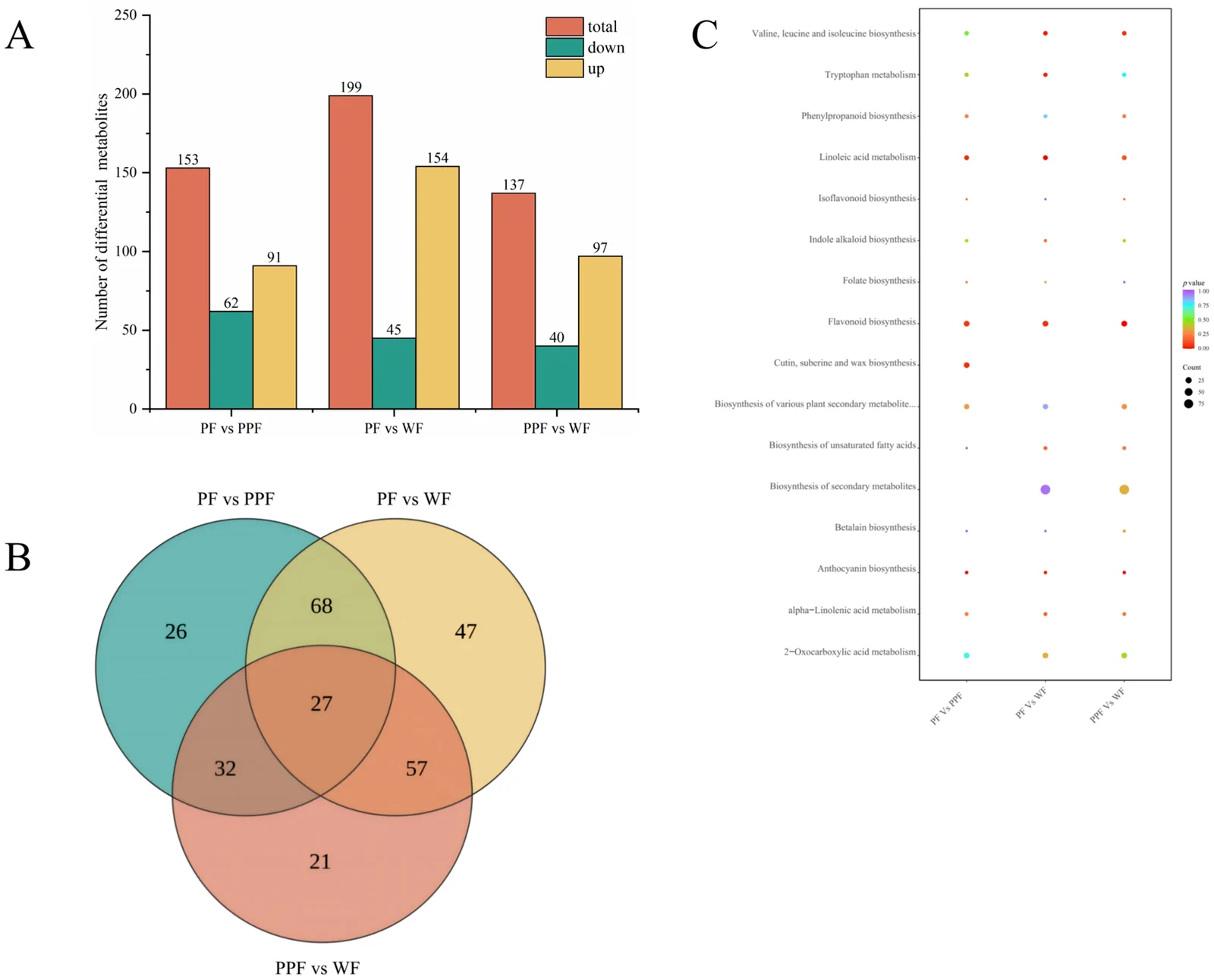
Differentially accumulated metabolites (DAMs) among PF, PPF, and WF. **(A)** Numbers of DAMs with higher or lower abundance in each pairwise comparison. **(B)** Venn diagram showing shared and comparison-specific DAMs. **(C)** Top 16 KEGG pathways enriched among DAMs. The y-axis shows KEGG pathways, and the x-axis shows the pairwise comparison groups.

The three cultivars displayed pink (PF), pale-pink (PPF), and white (WF) petals (**Figure 1A**). Widely targeted metabolomic profiling detected 689 metabolites assigned to 16 classes. Flavonoids were the largest class (24.5%), followed by phenolic acids (16.3%) and lipids (11.8%). Principal component analysis separated the three cultivar groups, with PC1 and PC2 explaining 45.32% and 30.40% of the variance, respectively (**Figure 2A**). The PF *vs.* PPF comparison contained 153 DAMs (91 higher and 62 lower in PF), PF *vs.* WF contained 199 DAMs (154 higher and 45 lower in PF), and PPF *vs.* WF contained 137 DAMs (40 higher and 97 lower in PPF) (**Figure 3A**). Twenty-seven DAMs were shared among all three comparisons (**Figure 3B**). KEGG annotation highlighted phenylpropanoid biosynthesis, flavone and flavonol biosynthesis, and anthocyanin biosynthesis, supporting a major contribution of flavonoid metabolism to the observed color differences.

### Comparative flavonoid metabolomics of three Chinese peony cultivars

**Figure 4:**
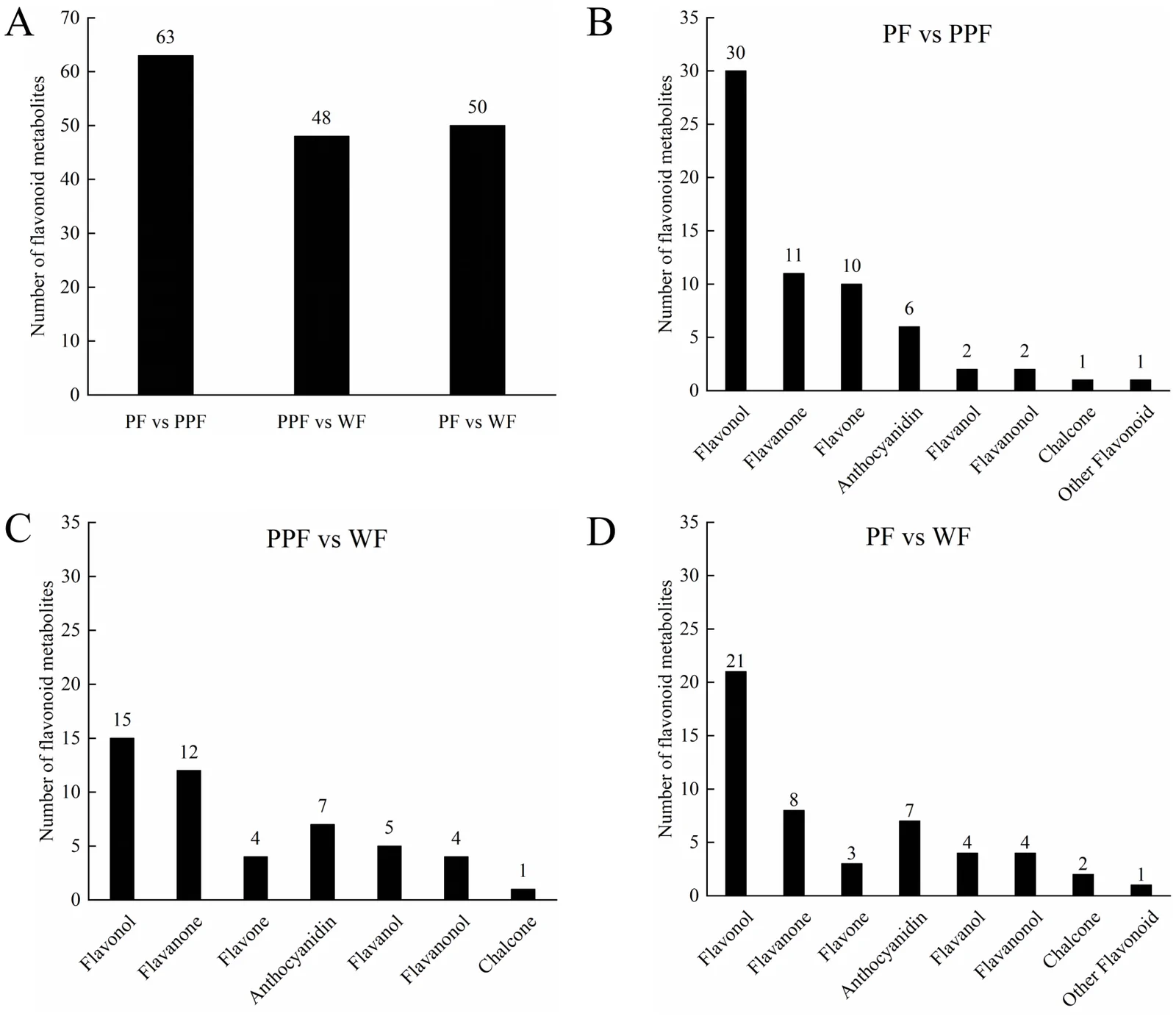
Differentially accumulated flavonoids in the three cultivars. **(A)** Total flavonoid abundance. **(B–D)** Differentially accumulated flavonoid subclasses in PF *vs.* PPF, PPF *vs.* WF, and PF *vs.* WF, respectively. PF, cv. ‘Exclusive Memory’ (PF), cv. ‘Salad’ (PPF), and cv. ‘Duchess’ (WF).

KEGG enrichment of the DAMs identified pathways related to anthocyanin biosynthesis, flavone and flavonol biosynthesis, phenylpropanoid biosynthesis, tryptophan metabolism, and several alkaloid pathways (**Figure 3C**). Among the 689 detected metabolites, 169 were flavonoids. These comprised seven major subclasses, of which flavonols (47.90%), flavones (19.50%), and flavanones (14.20%) were the most abundant (**Figure 2B**). We identified 63 differentially accumulated flavonoids (DAFs) in PF *vs.* PPF (22 higher and 41 lower in PF), 50 in PF *vs.* WF (26 higher and 24 lower in PF), and 48 in PPF *vs.* WF (30 higher and 18 lower in PPF). The 63 DAFs in PF *vs.* PPF comprised 30 flavonols, 11 flavanones, 10 flavones, six anthocyanins, two flavanols, two flavanonols, one chalcone, and one other flavonoid (**Figures 4A, B**). The corresponding compositions for PPF *vs.* WF and PF *vs.* WF are shown in **Figures 4C, D**. Total flavonoid abundance did not differ significantly between PF and PPF (*P* > 0.05), whereas both colored cultivars differed significantly from WF (*P* < 0.05).

### Comparative anthocyanin profiling of three Chinese peony cultivars

**Figure 5:**
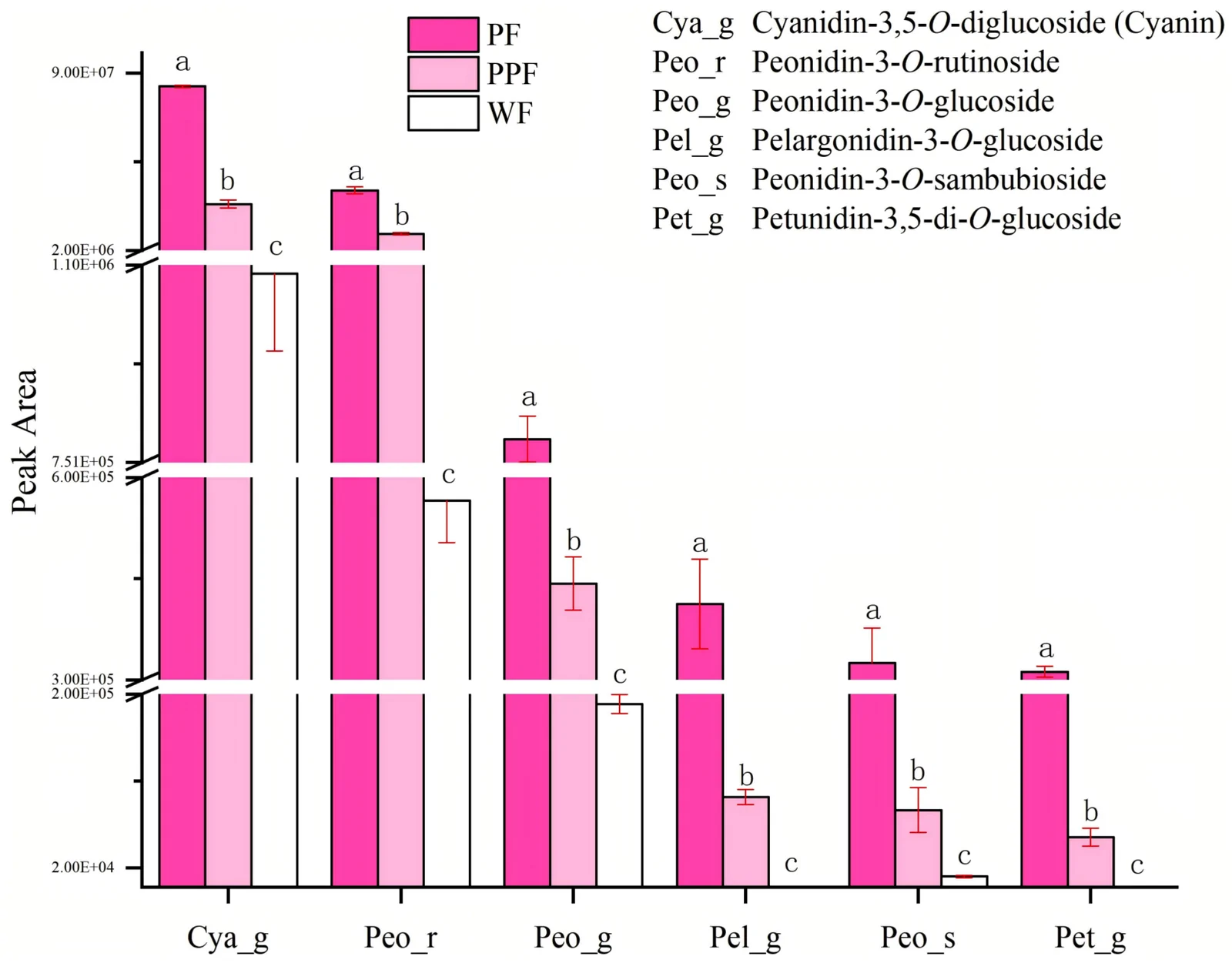
Relative peak areas of six anthocyanins in PF, PPF, and WF petals. Values are shown for three biological replicates. PF, cv. ‘Exclusive Memory’ (PF), cv. ‘Salad’ (PPF), and cv. ‘Duchess’ (WF).

Six anthocyanins were detected across the three cultivars, with the highest overall abundance in PF and the lowest in WF (**Figure 5**). Cyanidin-3,5-*O*-diglucoside (cyanin) was the predominant anthocyanin and accounted for a substantial fraction of the anthocyanin signal. The total anthocyanin signal in PF petals was 3.25-fold that in PPF and 60.37-fold that in WF (**Figure 1B**). These total-content ratios should not be conflated with the fold changes of individual metabolites. Specifically, cyanin abundance was 76.85-fold higher in PF than in WF and 23.10-fold higher in PPF than in WF. Peonidin-3-*O*-rutinoside was another abundant anthocyanin, with levels 3.09-fold higher in PF than in PPF and 56.31-fold higher in PF than in WF. Thus, the pink-to-white gradient was associated with both a reduction in total anthocyanin abundance and changes in the relative abundance of individual cyanidin- and peonidin-derived glycosides.

**Figure 6:**
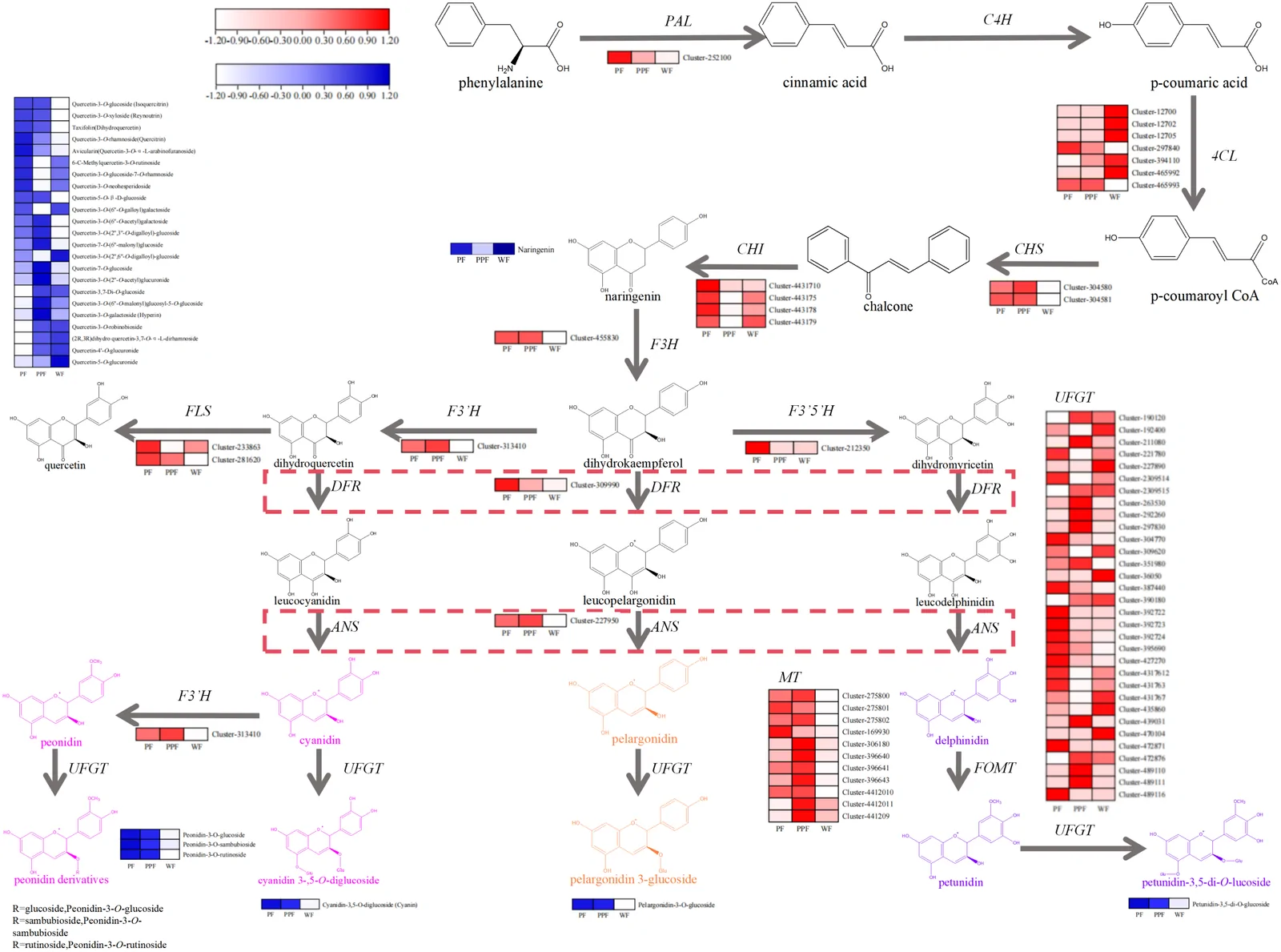
Integrated view of metabolites and transcripts in the phenylpropanoid–flavonoid pathway in PF, PPF, and WF petals. Colors indicate the mean abundance or expression value from three biological replicates. *PAL*, phenylalanine ammonia-lyase; *C4H*, cinnamate 4-hydroxylase; *4CL*, 4-coumarate:CoA ligase; *CHS*, chalcone synthase; *CHI*, chalcone isomerase; *F3H*, flavanone 3-hydroxylase; *F3’H*, flavonoid 3’-hydroxylase; *F3’5’H*, flavonoid 3’,5’-hydroxylase; *DFR*, dihydroflavonol 4-reductase; *ANS*, anthocyanidin synthase; *FLS*, flavonol synthase; *FOMT*, flavonoid *O*-methyltransferase; *UFGT*, UDP-glucose:flavonoid 3-*O*-glucosyltransferase.

Mapping metabolites and DEGs to the phenylpropanoid-flavonoid pathway showed coordinated differences in anthocyanin-related metabolites and transcripts, including UDP-glycosyltransferase candidates (**Figure 6**). These associations nominate biosynthetic steps for further study but do not by themselves demonstrate altered enzyme activity or direct causal regulation.

### Transcriptomic profiling and DEG analysis

RNA sequencing generated 61.1 Gb of clean data, with at least 6 Gb per library. Q30 values exceeded 91%, and the mean mapping rate was 93%.

**Figure 7:**
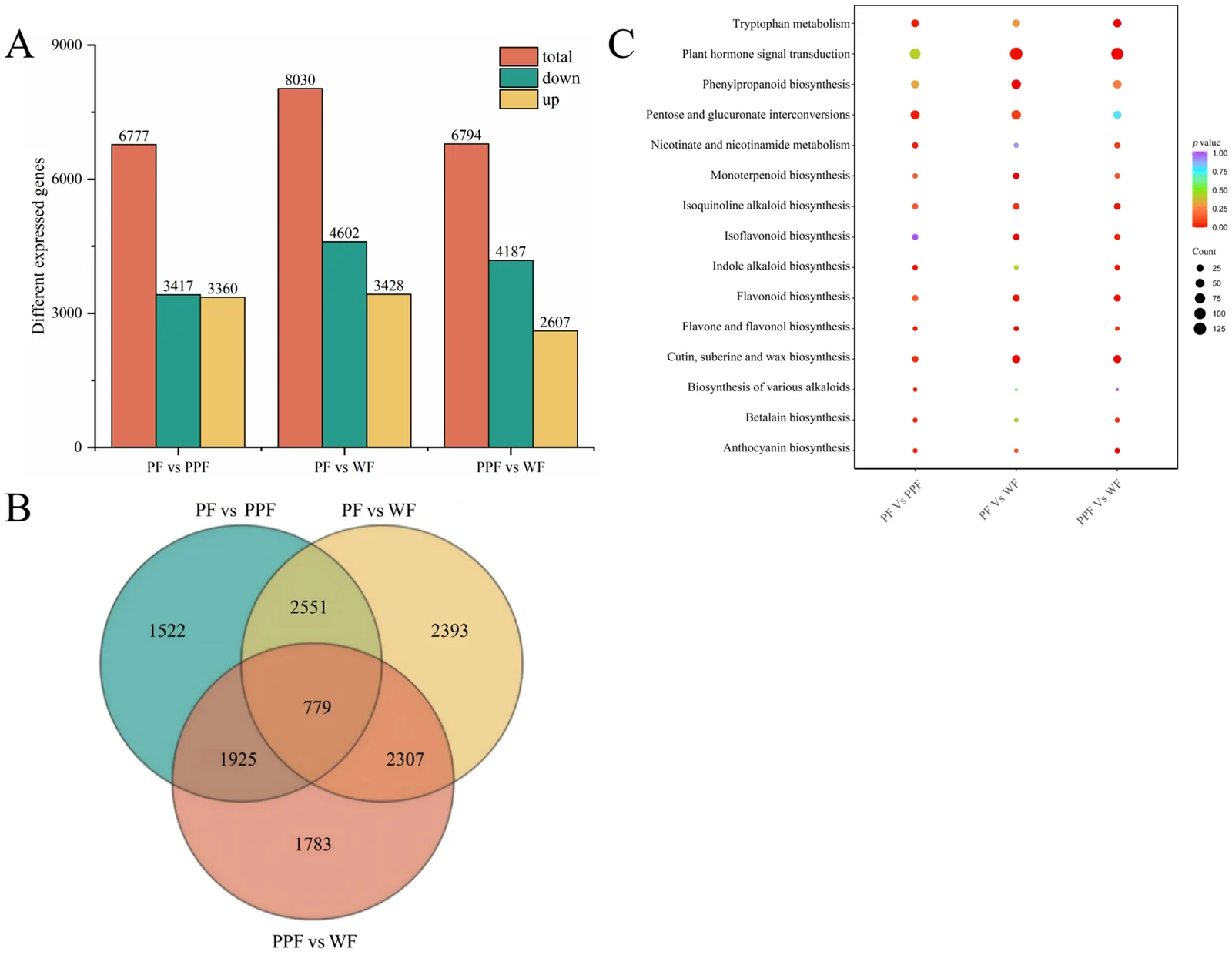
Differential gene expression analysis among PF, PPF, and WF peony petals. **(A)** Quantification of upregulated and downregulated differentially expressed genes (DEGs) in each pairwise comparison. **(B)** Venn diagram illustrating the 779 overlapping DEGs shared across the three comparisons. **(C)** KEGG pathway enrichment analysis of the identified DEGs. The y-axis and x-axis represent the enriched KEGG pathways and comparison groups, respectively.

A total of 75,549 genes were annotated. PCA separated PF, PPF, and WF while showing close clustering of the three biological replicates within each cultivar; PC1 and PC2 explained 26.22% and 20.95% of the variance, respectively (**Supplementary Figure 1**). PF *vs.* PPF contained 6,777 DEGs (3,360 upregulated and 3,417 downregulated in PF), PF *vs.* WF contained 8,030 DEGs (3,428 upregulated and 4,602 downregulated in PF), and PPF *vs.* WF contained 6,794 DEGs (2,607 upregulated and 4,187 downregulated in PPF) (**Figure 7A**). A total of 779 DEGs were shared among the three comparisons (**Figure 7B**). GO enrichment was dominated by biological-process terms, followed by molecular-function and cellular-component terms (**Supplementary Figure 2**). KEGG analysis highlighted 15 pathways, including flavonoid biosynthesis (**Figure 7C**). These results identify expression programs associated with cultivar color differences but do not establish that all enriched processes directly control pigmentation.

### Candidate genes associated with flavonoid and anthocyanin biosynthesis

**Figure 8:**
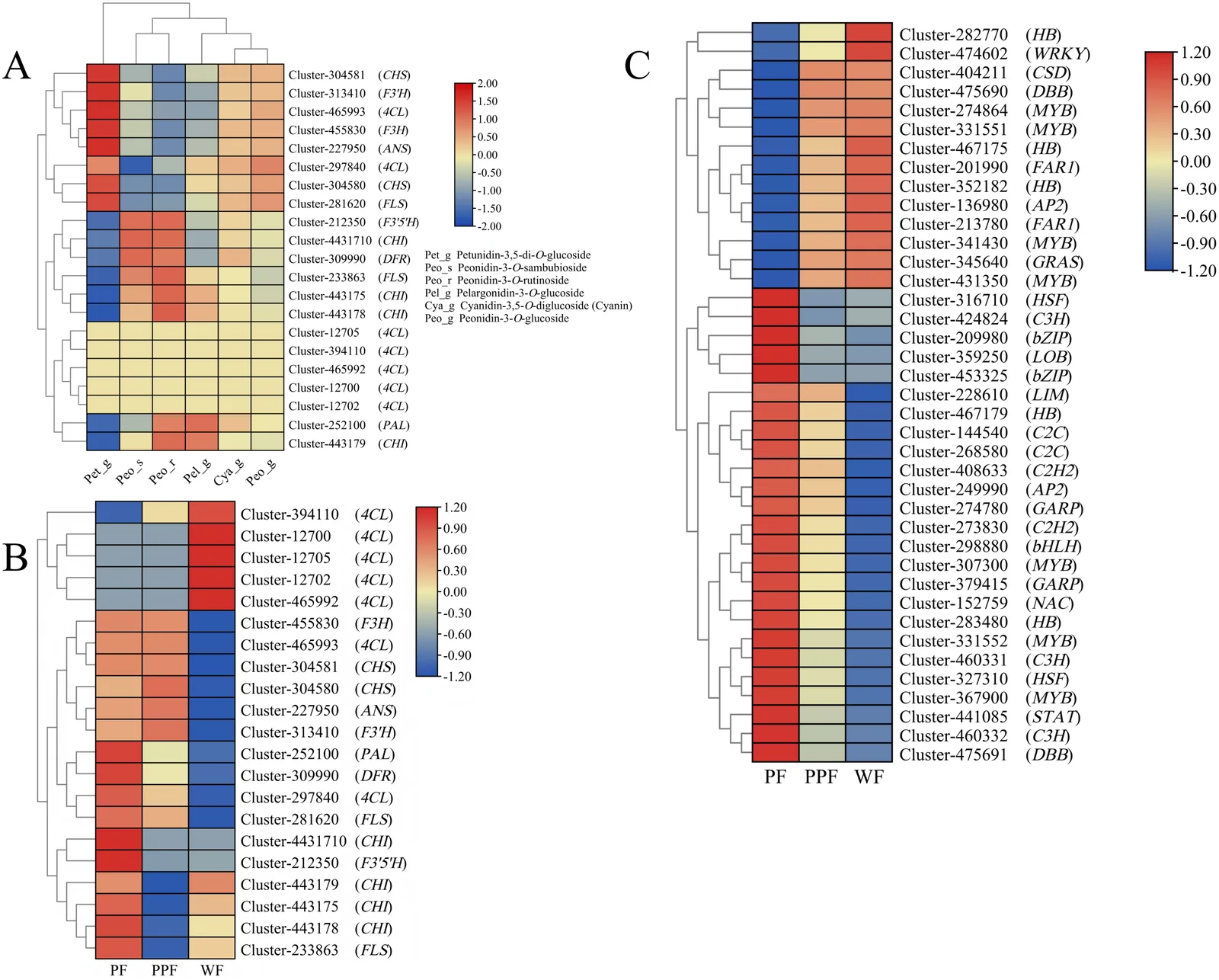
Expression profiles and correlation analysis of candidate genes associated with anthocyanin abundance. **(A)** Pearson-correlation heatmap associating the abundance of six individual anthocyanins with the expression of 21 structural genes in the flavonoid biosynthesis pathway. Heatmaps illustrating the relative expression of **(B)** the 21 structural genes across the three (P) lactiflora cultivars (PF, PPF, and WF) and **(C)** candidate transcription factors. For **(B, C)**, expression values (FPKM) were row-scaled, with the color gradient from blue to red representing low to high relative expression levels. For **(A)**, the color scale denotes the correlation coefficient.

DEGs were identified in flavonoid biosynthesis (ko00941), phenylpropanoid biosynthesis (ko00940), and flavone and flavonol biosynthesis (ko00944). By conducting a correlation analysis between anthocyanin abundance (six individual compounds and total content) and pathway-related DEGs, 21 candidate structural genes were prioritized: *PAL* (Cluster-252100); *4CL* (Cluster-12700, Cluster-12702, Cluster-12705, Cluster-297840, Cluster-394110, Cluster-465992, and Cluster-465993); *FLS* (Cluster-233863 and Cluster-281620); *CHS* (Cluster-304580 and Cluster-304581); *CHI* (Cluster-4431710, Cluster-443175, Cluster-443178, and Cluster-443179); *F3H* (Cluster-455830); *F3’H* (Cluster-313410); *F3’5’H* (Cluster-212350); *DFR* (Cluster-309990); and *ANS* (Cluster-227950) (**Figures 8A, B**). Among 317 annotated transcription factors, 157 were differentially expressed. Using |r| > 0.80 as a stringent prioritization threshold, 53 transcription factors were associated with anthocyanin abundance. Positively correlated candidates included *MYB* genes Cluster-307300 (r = 0.96), Cluster-331552 (r = 0.97), and Cluster-367900 (r = 0.98), and *bHLH* genes Cluster-253660 (r = 0.87) and Cluster-298880 (r = 0.98). Negatively correlated candidates included *bHLH* Cluster-170980 (r = -0.81), *WRKY* Cluster-474602 (r = -0.91), and *MYB* genes Cluster-341430 (r = -0.913), Cluster-431350 (r = -0.96), and Cluster-274864 (r = -0.97) (expression profiles are shown in **Figure 8C**). All reported correlations had *P* < 0.01. These genes are described as candidates.

### Anthocyanin-associated modules and candidate hub genes identified by WGCNA

**Figure 9:**
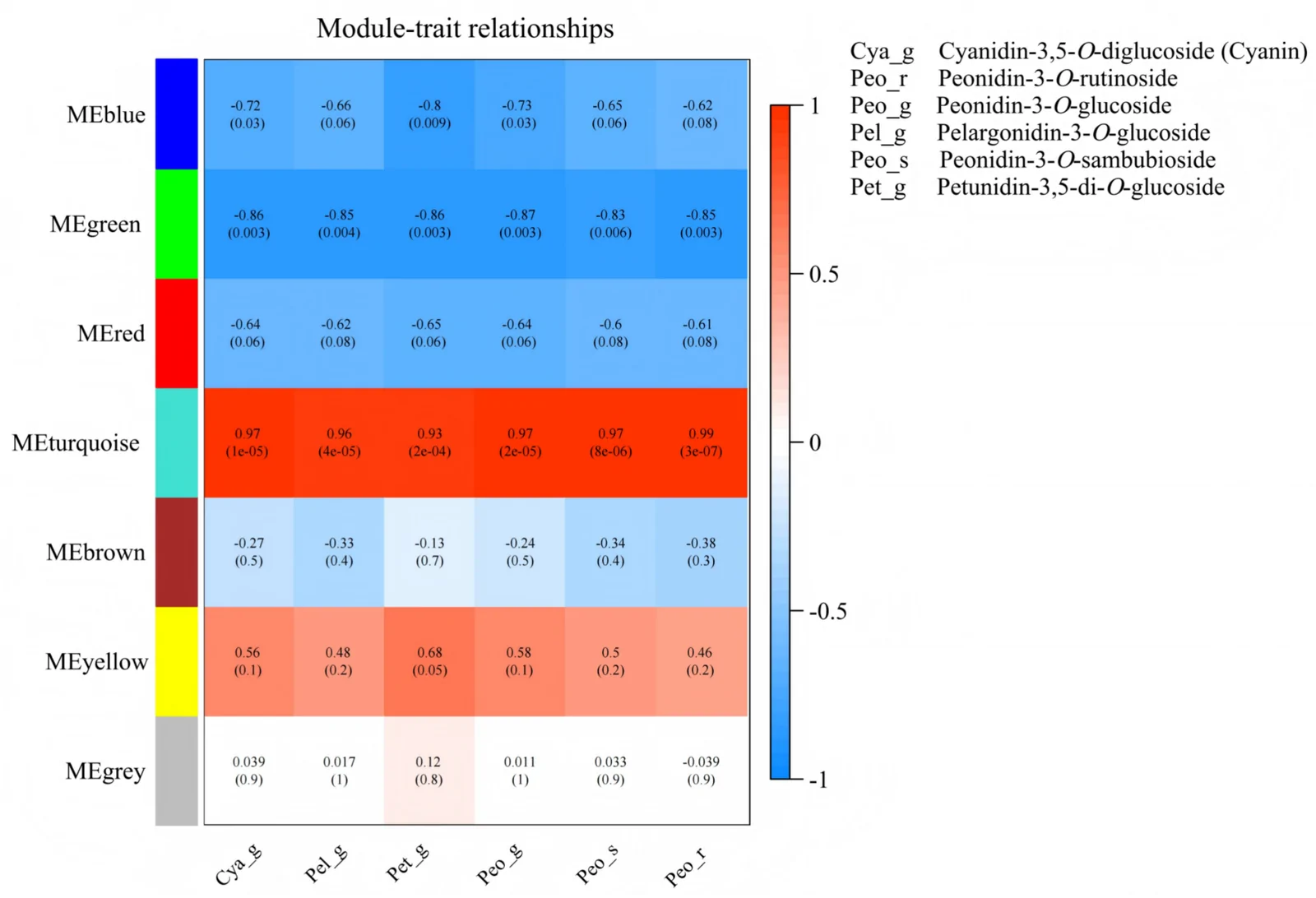
WGCNA module-trait relationships. Rows represent co-expression modules and columns represent anthocyanin traits or cultivar groups. Each cell shows the correlation coefficient and corresponding *P* value. The number of genes in each module is indicated alongside the module name.

WGCNA was used to examine co-expression patterns associated with anthocyanin abundance. A soft-thresholding power of β = 18 produced an approximate scale-free topology (R² > 0.85). Hierarchical clustering and dynamic tree cutting identified seven co-expression modules (**Figure 9**). The turquoise module showed the strongest positive association with anthocyanin abundance and was selected for downstream analysis.

**Figure 10:**
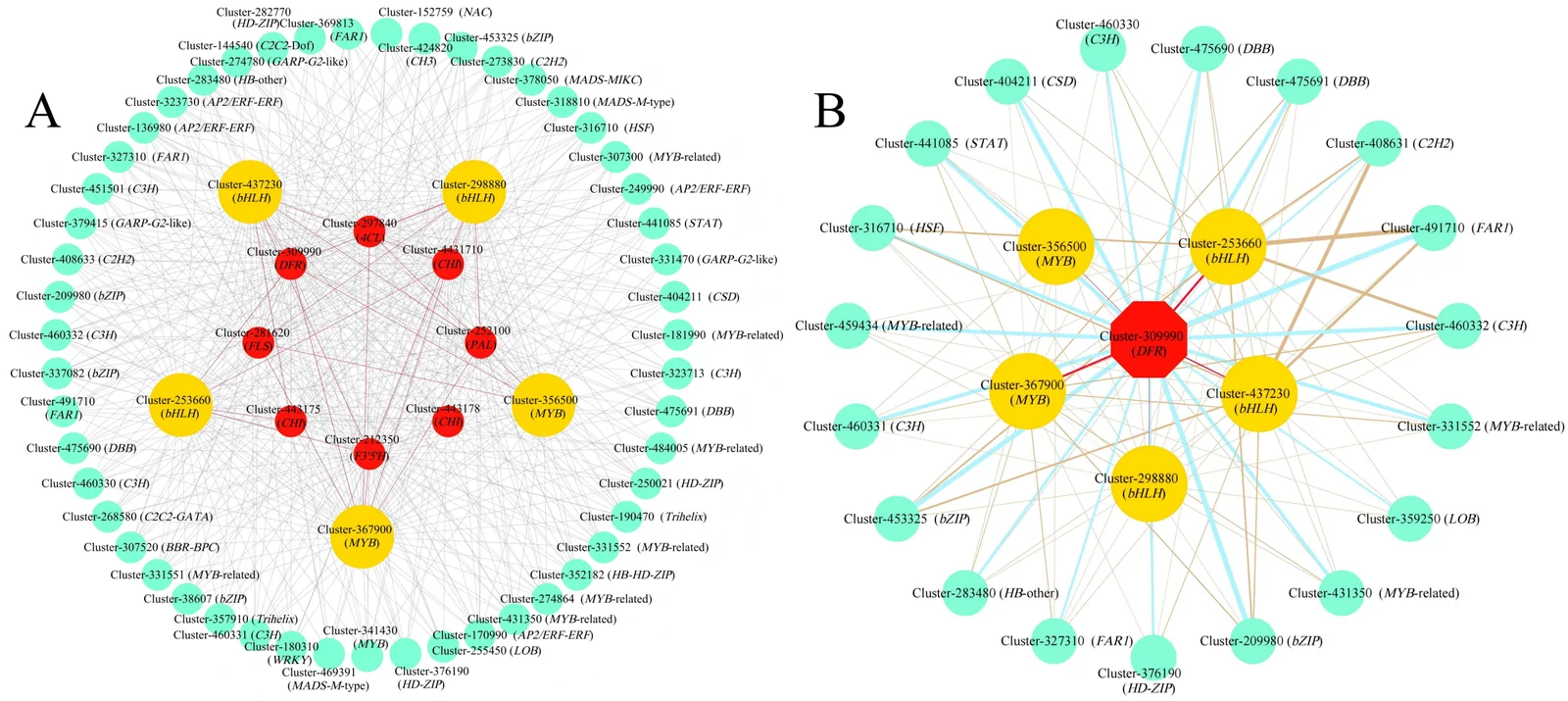
Candidate co-expression networks associated with anthocyanin biosynthesis. **(A)** Scaled co-expression network involving transcription factors, structural genes, and anthocyanin traits within the turquoise module after stringent filtering based on topological overlap matrix (TOM) weights, module membership, and gene significance. **(B)** *DFR*-centered subnetwork delineating 24 strongly correlated transcription factors. Edges represent co-expression or correlation links and do not infer direct biochemical regulation.

The turquoise module contained 4,770 genes, including 108 transcription factors and eight flavonoid-pathway structural genes. Filtering by TOM weight > 0.24 retained a 107 gene subnetwork, which was further prioritized using |module membership| > 0.80 and |gene significance| > 0.20. The resulting network included 91 transcription factors from 33 families and eight structural genes, together with other co-expressed genes (**Figure 10A**). The transcription factor families included *NAC*, *C3H*, *C2H2*, *FAR1*, *WRKY*, *bHLH*, *bZIP*, *MYB*, and *MYB*-related genes (detailed statistical classifications are provided in **Supplementary Table 2**).

The eight structural genes in the prioritized network were *PAL* (Cluster-252400), *4CL* (Cluster-297840), *CHI* (Cluster-443175, Cluster-443178, and Cluster-4431710), *FLS* (Cluster-281620), *F3’5’H* (Cluster-212350), and *DFR* (Cluster-309990). A *DFR*-centered correlation subnetwork contained 24 strongly associated transcription factors (**Figure 10B**). Candidate hubs included *MYB* genes Cluster-367900 and Cluster-356500 and *bHLH* genes Cluster-437230, Cluster-298880, and Cluster-253660. *NAC* (Cluster-152759), *WRKY* (Cluster-180310), *AP2* (Cluster-249990), *C2H2* (Cluster-268580), and *FAR1* (Cluster-327310) were also positively associated with anthocyanin abundance. These nodes represent high-priority candidates for functional testing, not confirmed direct regulators of *DFR* or anthocyanin biosynthesis.

### Expression analysis of genes through qRT-PCR

**Figure 11:**
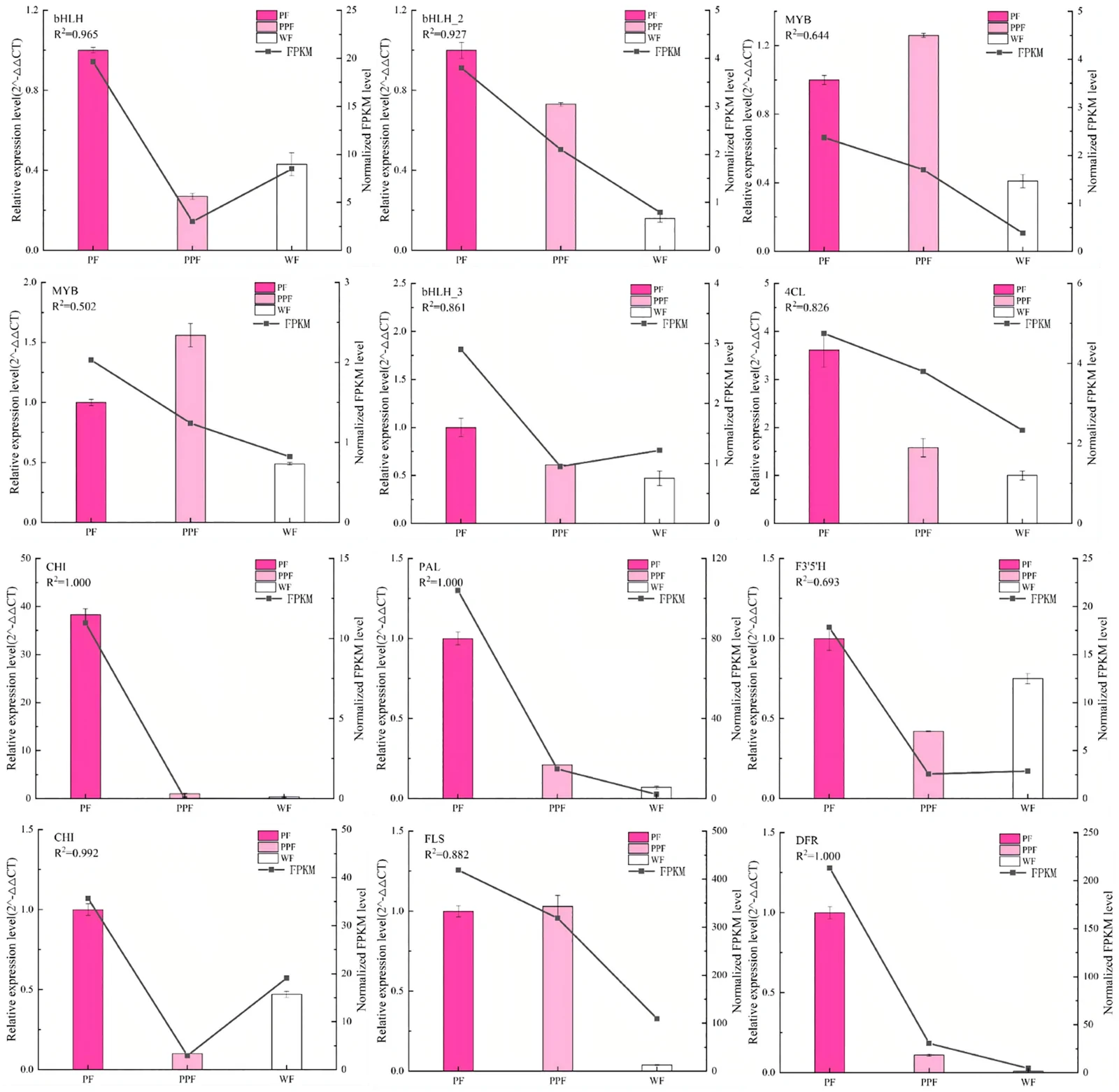
Comparison of qRT-PCR and RNA-seq expression patterns for 12 selected genes: *DFR* (Cluster-309990), *MYB* (Cluster-356500 and Cluster-367900), *4CL* (Cluster-297840), *CHI* (Cluster-443175 and Cluster-443178), *PAL* (Cluster-252400), *F3’5’H* (Cluster-212350), *FLS* (Cluster-281620) and *bHLH* (Cluster-437230, Cluster-298880, and Cluster-253660). Relative expression was calculated using the 2^^−ΔΔCt^ method. Statistical differences among groups were determined by one-way ANOVA followed by Tukey’s HSD (*P* < 0.05). Data are presented as means ± SD of three biological replicates, each measured in three technical reactions.

qRT-PCR was used to examine 12 selected genes: *DFR* (Cluster-309990), *MYB* (Cluster-356500 and Cluster-367900), *4CL* (Cluster-297840), *CHI* (Cluster-443175 and Cluster-443178), *PAL* (Cluster-252400), *F3’5’H* (Cluster-212350), *FLS* (Cluster-281620) and *bHLH* (Cluster-437230, Cluster-298880, and Cluster-253660). For all 12 genes, the direction of expression change across PF, PPF, and WF was consistent with the RNA-seq data (**Figure 11**), supporting the reproducibility of the expression profiles. This validation confirms expression trends, thereby confirming the reliability of the transcriptomic findings.

## Discussion

### Key anthocyanin metabolites associated with Chinese peony flower color

Flower color can be generated by several pigment groups, notably betalains, carotenoids, and flavonoids. Carotenoids contribute yellow, orange, and red hues in many crops (Wang et al., 2025, Wang et al., 2025; Xing et al., 2022; Zhang et al., 2015), whereas anthocyanins are the principal red-to-blue flavonoid pigments in most flowering plants; betalain pigmentation is regulated through a distinct system (Lloyd et al., 2017). In the three *P. lactiflora* cultivars examined here, the close correspondence between petal color intensity and anthocyanin abundance indicates that anthocyanins are the dominant pigment class underlying the pink-to-white gradient.

The total anthocyanin signal decreased stepwise from PF to PPF to WF, with PF containing 3.25-fold more than PPF and 60.37-fold more than WF. Similar associations between anthocyanin abundance and flower-color intensity have been reported in *Camellia japonica*, *miniature rose*, and *Impatiens hybrida* (Fu et al., 2021; Lu et al., 2021; Tian et al., 2025; Lim et al., 2020). Nevertheless, total abundance alone does not fully explain hue because anthocyanin structure, glycosylation, acylation, co-pigments, vacuolar pH, and metal ions can modify the visible phenotype. Our data therefore support a quantitative contribution of anthocyanins while leaving these additional physicochemical factors unresolved.

Cyanin was the predominant detected anthocyanin, and its compound-specific fold change was larger than the fold change in total anthocyanin content. This distinction resolves the apparent numerical discrepancy among sections of the original manuscript: the 3.25- and 60.37-fold values refer to the summed anthocyanin signal, whereas the 76.85- and 23.10-fold values refer specifically to cyanin. Cyanin and peonidin-derived glycosides were strongly depleted in WF, suggesting that both cyanidin- and peonidin-branch fluxes contribute to pink coloration. Glycosylation can increase anthocyanin stability and facilitate vacuolar accumulation, and a *P. lactiflora* glycosyltransferase has previously been shown to modify anthocyanins during petal coloration (Li et al., 2022). However, targeted standards, absolute quantification, and perturbation of individual glycosyltransferases will be required to establish the causal contribution of each compound.

### Structural-gene candidates associated with anthocyanin accumulation

Studies have demonstrated that the differential expression of structural genes, primarily *DFR* and *ANS*, constitutes the key molecular basis for anthocyanin pigmentation (Xia et al., 2022). The expression patterns of pathway genes suggest that reduced flux through late anthocyanin biosynthesis contributes to the paler phenotypes. *DFR* is a key branch-point enzyme that converts dihydroflavonols to leucoanthocyanidins; *ANS* then produces anthocyanidins, and glycosyltransferases stabilize the products as anthocyanins. Changes in these late steps have been associated with petal pigmentation in *Camellia japonica*, *Brassica napus*, *Rhododendron pulchrum*, and *Impatiens hybrida* (Fu et al., 2021; Hao et al., 2022; Xia et al., 2022; Tian et al., 2025; Zhou et al., 2017). In our dataset, *DFR* (Cluster-309990) was more highly expressed in PF and was positively associated with anthocyanin abundance. This pattern is mechanistically consistent with increased precursor conversion toward anthocyanins, provided that substrate supply and downstream *ANS* and glycosyltransferase activities are not limiting. We therefore identify *DFR* as a strong candidate associated with color intensity, but the present data do not establish that DFR alone determines the phenotype.

### Candidate transcriptional network associated with anthocyanin biosynthesis

*MYB*, *bHLH*, and *WD40* proteins commonly form the *MBW* regulatory complex that controls anthocyanin structural genes, and *WRKY* and other transcription factors can interact with or act alongside this core system (Jian et al., 2019; Lloyd et al., 2017; Wang et al., 2023; Feng et al., 2020). In this study, 53 transcription factors were strongly correlated with anthocyanin abundance. The opposing expression patterns of positively and negatively correlated *MYB*, *bHLH*, and *WRKY* candidates could contribute to the graded expression of structural genes across PF, PPF, and WF. However, the labels “positive” and “negative” refer only to correlation direction and should not be interpreted as proof of transcriptional activation or repression.

WGCNA provided an independent network-level prioritization. The turquoise module was positively associated with anthocyanin abundance, and stringent TOM, module-membership, and gene-significance thresholds reduced this module to a focused candidate network. Transparent reporting of trait definition and filtering thresholds is important because WGCNA results depend on sample structure, trait measurement, and network parameters (Langfelder and Horvath, 2008; Hong et al., 2015; Bai et al., 2025; Katsumoto et al., 2007). Liu et al. (2026), for example, showed that using a dynamic fiber-elongation-rate trait improved the biological interpretation of cotton WGCNA modules. In our analysis, quantitative anthocyanin abundance served as the module-associated trait.

The *DFR*-centered subnetwork prioritized two *MYB* genes (Cluster-367900 and Cluster-356500) and three *bHLH* genes (Cluster-437230, Cluster-298880, and Cluster-253660), together with *NAC*, *WRKY*, *AP2*, *C2H2*, and *FAR1* candidates. Their co-expression with *DFR* is compatible with coordinated pathway control, but direct binding and transcriptional effects were not tested. A plausible working model is that these transcription factors modulate *DFR* and other late biosynthetic genes, thereby influencing the amount and composition of stable anthocyanins. This model should be evaluated using promoter-binding assays, dual-luciferase reporter assays, transient expression or virus-induced gene silencing, stable overexpression or knockout lines, and targeted metabolite measurements.

## Conclusion

Integrated metabolomic and transcriptomic analyses associated the pink-to-white color gradient in three *P. lactiflora* cultivars with progressive decreases in total anthocyanins and with pronounced changes in cyanidin-3,5-*O*-diglucoside. The analyses prioritized 21 structural-gene candidates, 53 anthocyanin-correlated transcription factors, and a turquoise co-expression module containing a *DFR*-centered candidate network. 12 selected genes showed qRT-PCR expression trends consistent with RNA-seq. These findings provide a testable set of candidate metabolites and genes for future studies of peony flower coloration. Because the proposed network is based on three cultivars, one developmental stage, and correlation-based analyses, functional experiments are required before any candidate can be considered a confirmed regulator or breeding target.

## Data Availability

The data presented in the study are deposited in the National Genomics Data Center (NGDC) repository, accession number CRA048652.
