# Supplementary material for: Integrated metabolome and transcriptome analysis reveals the potential mechanism of anthocyanin biosynthesis in Paeonia lactiflora Pall. flowers
Source: Front Plant Sci. 2026 Aug 31;17:1907085. doi: 10.3389/fpls.2026.1907085 (PMC13572631; doi:10.3389/fpls.2026.1907085)
Supplement: Supplementary file 1 [file DataSheet1.docx]

***Supplementary Material***

**Supplementary Table 1 Primers used in qRT-PCR**

| Gene ID |  | sequence（5'-3'） | product size |
| --- | --- | --- | --- |
| Cluster-297840 | F | GTTTTGTGCTCACTGTTTGTGA | 110bp |
|  | R | CAAGGTCCAGCCACCATTCTA |  |
| Cluster-212350 | F | TCCAACACCGAGTTCCATCC | 112bp |
|  | R | GAACCACAGGTAGGGGACTG |  |
| Cluster-443178 | F | CTCAACACCACCTCATCGTCA | 126bp |
|  | R | GTCTGTCCATCAACGAGGGG |  |
| Cluster-443175 | F | TGTGTGGCCTACTGGAAATCT | 192bp |
|  | R | TACTGCATTCCCAGCTTCGG |  |
| Cluster-309990 | F | CCAGAAGTACTGATCGACCCC | 185bp |
|  | R | AGAGGACATGTTTGTCGAAGC |  |
| Cluster-281620 | F | CCGATTTGTCCACCCTCACA | 160bp |
|  | R | ACGCTCCTGTACTTGCCATT |  |
| Cluster-252400 | F | GCCGTTCTTCACCGGACTAC | 108bp |
|  | R | AACAAGTTTTGGATGAGGCCC |  |
| Cluster-356500 | F | AGATCCATCACCATCGGAGC | 139bp |
|  | R | TTTGAGCATCACAAGCTGGG |  |
| Cluster-298880 | F | CGATTGCCAAACCACCACAT | 159bp |
|  | R | CCTACGTTGAAGCCCAGGTC |  |
| Cluster-367900 | F | CTTCAATCCGACGCAAACGG | 149bp |
|  | R | TCATCCCTGGACTCACGCTA |  |
| Cluster-437230 | F | TAAGTATGTGCCCACTGCTGAT | 119bp |
|  | R | CGGGCAAGCTCATGGATGG |  |
| Cluster-253660 | F | ATGGGTCACGGCAAAATGTG | 149bp |
|  | R | CACAGAAAACTGCCCACGAA |  |
| UBQ10 | F | CGGAGGACAAGATGGAGTGT | 127bp |
|  | R | TCCAAGACAAGGAAGGCATC |  |

**Supplementary Table 2 Statistical distribution and classification of differentially expressed transcription factor (TF) genes related to anthocyanin biosynthesis in P. lactiflora petals**

| ID | TF-Family |  | PF | PPF | WF | PF vs WF | | | PPF vs WF | | | PF vs PPF | | |
| --- | --- | --- | --- | --- | --- | --- | --- | --- | --- | --- | --- | --- | --- | --- |
| Gene ID | TFs name | Correlation with total anthocyanin | PF | PPF | WF | Log2FC | Fdr | Type | Log2FC | Fdr | Type | Log2FC | Fdr | Type |
| Cluster-136980 | *AP2/ERF-ERF* | -0.949 | 4.01 | 8.69 | 11.65 | 1.52 | 4.70E-10 | up | 0.42 | 1.37E-01 | false | 1.09 | 1.91E-05 | up |
| Cluster-144540 | *C2C2-Dof* | 0.947 | 73.66 | 44.13 | 20.24 | -1.88 | 2.26E-23 | down | -1.13 | 1.54E-14 | down | -0.75 | 3.18E-05 | false |
| Cluster-152759 | *NAC* | 0.956 | 26.74 | 11.85 | 4.70 | -2.46 | 2.04E-03 | down | -1.38 | 1.75E-01 | false | -1.09 | 1.75E-02 | down |
| Cluster-201990 | *FAR1* | -0.902 | 0.12 | 1.74 | 2.73 | 4.57 | 3.61E-03 | up | 0.65 | 5.04E-01 | false | NA | NA | NA |
| Cluster-209980 | *bZIP* | 0.914 | 7.64 | 0.76 | 0.30 | -4.67 | 1.23E-08 | down | NA | NA | NA | -3.34 | 3.04E-07 | down |
| Cluster-213780 | *FAR1* | -0.943 | 0.29 | 1.09 | 1.52 | 2.36 | 7.47E-05 | up | 0.48 | 4.13E-01 | false | 1.87 | 5.13E-03 | up |
| Cluster-228610 | *LIM* | 0.919 | 61.77 | 42.53 | 10.37 | -2.58 | 9.66E-55 | down | -2.04 | 8.14E-25 | down | -0.55 | 1.13E-03 | false |
| Cluster-249990 | *AP2/ERF-ERF* | 0.958 | 10.30 | 4.97 | 0.72 | -3.89 | 4.86E-15 | down | -2.82 | 1.00E-07 | down | -1.08 | 9.50E-03 | down |
| Cluster-268580 | *C2C2-GATA* | 0.965 | 51.52 | 33.93 | 17.88 | -1.56 | 1.99E-24 | down | -0.94 | 1.53E-09 | false | -0.63 | 5.67E-08 | false |
| Cluster-273830 | *C2H2* | 0.934 | 5.85 | 1.95 | 0.02 | -7.85 | 6.05E-08 | down | -6.28 | 2.24E-04 | down | -1.58 | 1.74E-02 | down |
| Cluster-274780 | *GARP-G2*-like | 0.932 | 34.07 | 25.98 | 15.60 | -1.15 | 5.77E-16 | down | -0.74 | 6.50E-05 | false | -0.41 | 4.55E-02 | false |
| Cluster-274864 | *MYB*-related | -0.970 | 0.59 | 29.33 | 35.91 | 5.94 | 8.34E-17 | up | 0.27 | 5.40E-01 | false | 5.66 | 8.60E-13 | up |
| Cluster-282770 | *HB-KNOX* | -0.912 | 8.13 | 11.54 | 16.76 | 1.02 | 9.03E-07 | up | 0.53 | 5.28E-04 | false | 0.48 | 4.07E-02 | false |
| Cluster-283480 | *HB*-other | 0.932 | 15.47 | 3.15 | 0.19 | -6.55 | 1.34E-09 | down | -4.26 | 3.36E-02 | down | -2.29 | 6.73E-03 | down |
| Cluster-298880 | *bHLH* | 0.976 | 3.80 | 2.10 | 0.79 | -2.41 | 7.56E-08 | down | -1.68 | 7.04E-04 | down | -0.74 | 8.19E-02 | false |
| Cluster-307300 | *MYB*-related | 0.953 | 22.14 | 11.85 | 5.35 | -2.03 | 9.35E-11 | down | -1.14 | 7.76E-04 | down | -0.90 | 6.69E-03 | false |
| Cluster-316710 | *HSF* | 0.918 | 15.05 | 6.80 | 7.36 | -1.03 | 4.37E-06 | down | 0.19 | 6.15E-01 | false | -1.23 | 9.44E-08 | down |
| Cluster-327310 | *FAR1* | 0.969 | 2.96 | 1.00 | 0.23 | -3.74 | 6.41E-08 | down | NA | NA | NA | -1.58 | 7.04E-03 | down |
| Cluster-331551 | *MYB*-related | -0.913 | 0.00 | 2.65 | 3.16 | 8.15 | 1.84E-07 | up | 0.25 | 7.62E-01 | false | 7.89 | 3.08E-07 | up |
| Cluster-331552 | *MYB*-related | 0.972 | 9.00 | 2.84 | 1.06 | -3.13 | 2.13E-05 | down | -1.45 | 1.91E-01 | false | -1.69 | 3.88E-04 | down |
| Cluster-341430 | *MYB* | -0.909 | 0.21 | 1.34 | 1.80 | 3.08 | 1.68E-02 | up | 0.43 | 6.36E-01 | false | 2.64 | 9.30E-02 | false |
| Cluster-345640 | *GRAS* | -0.953 | 6.06 | 12.55 | 13.91 | 1.18 | 3.00E-10 | up | 0.15 | 5.48E-01 | false | 1.02 | 9.32E-08 | up |
| Cluster-352182 | *HB-HD-ZIP* | -0.935 | 3.34 | 7.10 | 9.01 | 1.41 | 3.17E-06 | up | 0.34 | 2.46E-01 | false | 1.06 | 1.12E-03 | up |
| Cluster-359250 | *LOB* | 0.901 | 5.79 | 2.78 | 2.60 | -1.14 | 9.89E-03 | down | -0.09 | 9.04E-01 | false | -1.07 | 2.31E-02 | down |
| Cluster-367900 | *MYB* | 0.985 | 2.03 | 1.24 | 0.82 | -1.36 | 1.83E-02 | down | -0.63 | 4.06E-01 | false | -0.74 | 2.09E-01 | false |
| Cluster-379415 | *GARP-G2*-like | 0.939 | 9.28 | 3.56 | 0.81 | -3.54 | 8.45E-02 | false | -2.13 | 4.46E-01 | false | -1.42 | 2.31E-02 | down |
| Cluster-404211 | *CSD* | -0.939 | 0.00 | 48.75 | 50.56 | 12.27 | 1.06E-22 | up | 0.04 | 9.24E-01 | false | 12.22 | 4.56E-22 | up |
| Cluster-408633 | *C2H2* | 0.980 | 48.51 | 23.98 | 3.91 | -3.66 | 9.62E-47 | down | -2.63 | 2.78E-20 | down | -1.05 | 4.83E-17 | down |
| Cluster-424824 | *C3H* | 0.905 | 0.84 | 0.00 | 0.08 | -3.51 | 7.91E-02 | false | NA | NA | NA | -9.13 | 5.38E-11 | down |
| Cluster-431350 | *MYB*-related | -0.962 | 1.18 | 2.53 | 2.90 | 1.31 | 8.30E-04 | up | 0.29 | 5.10E-01 | false | 1.01 | 1.82E-02 | up |
| Cluster-441085 | *STAT* | 0.938 | 1.13 | 0.26 | 0.00 | -8.39 | 3.52E-08 | down | NA | NA | NA | -2.25 | 1.62E-02 | down |
| Cluster-453325 | *bZIP* | 0.950 | 7.58 | 0.00 | 0.00 | -10.17 | 1.01E-14 | down | NA | NA | NA | -10.08 | 2.17E-14 | down |
| Cluster-460331 | *C3H* | 0.962 | 12.03 | 2.54 | 0.53 | -4.63 | 3.49E-08 | down | -2.36 | 1.86E-01 | false | -2.28 | 3.20E-02 | down |
| Cluster-460332 | *C3H* | 0.922 | 10.94 | 0.95 | 0.00 | -9.53 | 7.06E-12 | down | NA | NA | NA | -3.55 | 7.86E-08 | down |
| Cluster-467175 | *HB-BELL* | -0.906 | 18.59 | 40.43 | 57.82 | 1.61 | 2.31E-07 | up | 0.51 | 2.92E-02 | false | 1.09 | 8.37E-05 | up |
| Cluster-467179 | *HB-BELL* | 0.904 | 9.54 | 3.31 | 0.00 | -11.66 | 5.78E-19 | down | -10.11 | 9.58E-15 | down | -1.56 | 3.12E-05 | down |
| Cluster-474602 | *WRKY* | -0.913 | 5.93 | 11.22 | 20.17 | 1.75 | 2.90E-09 | up | 0.84 | 1.45E-09 | false | 0.90 | 8.81E-03 | false |
| Cluster-475690 | *DBB* | -0.924 | 0.00 | 52.22 | 50.47 | 11.86 | 4.39E-21 | up | -0.06 | 8.90E-01 | false | 11.91 | 5.57E-21 | up |
| Cluster-475691 | *DBB* | 0.973 | 39.47 | 14.10 | 9.55 | -2.06 | 2.27E-15 | down | -0.57 | 2.19E-01 | false | -1.50 | 3.96E-09 | down |

**Figure S1 Global transcriptomic profiling of the three *P. lactiflora* cultivar groups (n = 3). Principal component analysis (PCA) illustrating the sample separation based on the transcriptomic dataset.**

**Figure S2 Gene Ontology enrichment of DEGs in PF *vs.* PPF, PF *vs*. WF, and PPF *vs.* WF. Enriched terms are grouped by biological process, molecular function, and cellular component.**
